# Effect of HHFNC therapy on organ oxygenation and brain metabolism in neonates receiving exchange transfusion

**DOI:** 10.3389/fped.2024.1381808

**Published:** 2024-05-31

**Authors:** Wanting Li, Ruming Ye, Binyan Xie, Xiaofang Deng, Dan Li, Ying Lin, Guanhong Wu, Xianghui Huang

**Affiliations:** Fujian Provincial Key Laboratory of Neonatal Diseases, Xiamen Children’s Hospital, Xiamen, China

**Keywords:** newborns, HHFNC, exchange transfusion therapy, organ oxygen saturation, brain metabolism

## Abstract

**Background:**

Exchange transfusion therapy is a complex and invasive procedure with a high risk coefficient. This method involves replacing the entire blood of a child with fresh blood with double circulating blood volume in a short period, typically in 1–2 h. This procedure can cause the body's internal environment to be unstable, which can put newborns under a lot of stress. This stress can lead to many, including abnormal laboratory biochemical examination, low or high blood pressure, and apnea. There is also the possibility of secondary infection and, in severe cases, cardiac arrest. This study investigated the effects of Humidified high-flow nasal cannula (HHFNC) ventilation on hemodynamic stability and oxygenation during exchange transfusion in neonates. Furthermore, the effects on brain metabolism and salivary cortisol during exchange transfusion were also analyzed.

**Methods:**

In this study, the control group consisted of 45 cases of children who underwent simple blood exchange between 1 May 2017, and 31 December 2019 control group. The observation group consisted of 33 cases of children who underwent blood exchange under HHFNC support between 1 January 2020, and 30 April 2022. The study compared various physiological parameters between the control and the observation group. These included blood gas analysis, pulmonary artery pressure, ejection fraction, invasive mean arterial pressure, heart rate, cerebral oxygenation, intestinal oxygenation, renal oxygenation, and duration of blood exchange. Furthermore, the study also compared the changes in brain metabolic and salivary cortisol indicators between the two groups of children.

**Results:**

The results did not reveal any significant difference in PH, PaO_2,_ and duration of blood exchange between the control and the observation group. However, the observation group's invasive mean arterial pressure, ejection fraction, cerebral oxygenation, intestinal oxygenation, and renal oxygenation were higher than those of the control group. Furthermore, compared with the control group, the pulmonary artery pressure, heart rate, and PaCO_2_ were lower in the observation group. There was a statistically significant difference between the two groups of children in the relevant clinical indicators (total bilirubin, hemoglobin, SPO_2_, etc.) after exchange transfusion. After 1 h of blood exchange and after blood exchange, the salivary cortisol levels of the observation group were lower than the control group. The difference was statistically significant. The NAA/Cho and Cho/Cr values of the two groups of children were also significantly different.

**Conclusion:**

During blood exchange, unstable hemodynamics substantially impact organ oxygenation. The results of this study suggest that HHFNC and specific ventilation pressure support can improve the respiratory rate and help maintain blood flow stability and organ oxygenation. This technique can also reduce adverse reactions caused by blood exchange, minimizing patient stress and reducing the impact on brain metabolism.

## 1 Introduction

Exchange transfusion therapy is used universally to treat neonatal hyperbilirubinemia. However, it has been reported to significantly impact neonatal biochemical indicators and the blood's internal environment (1). Neonatal hyperbilirubinemia is a common condition in newborns. The toxicity of bilirubin can lead to neonatal bilirubin encephalopathy. This can cause severe harm to infants, including hearing impairment, intellectual disability, and even cerebral palsy (2, 3). Due to the high energy demand, the basal ganglia region of the neonatal brain has a high oxygen consumption and active biochemical metabolism. This also makes this region vulnerable to damage from high bilirubin concentrations (4). The body may experience hemodynamic fluctuations during the process of blood exchange (5). In such cases, hemodynamic instability can affect organ oxygenation and stimulate the child to a certain extent. Through its constant warming and humidification function, Humidified high-flow nasal cannula oxygen therapy (HHFNC) can increase lung compliance, reduce resistance, promote alveolar expansion, reduce mucosal damage, and increase oxygen therapy comfort (6). To date, there has been limited research dedicated to maintaining organ oxygen saturation (brain tissue, intestinal tissue, and renal tissue) and brain metabolism during blood exchange. In our neonatal department, HHFNC is utilized to maintain stable blood flow and organ oxygenation during blood exchange. This approach helps reduce adverse reactions caused by blood exchange, minimizes stress in children, and lessens the impact on brain metabolism, leading to positive outcomes. The research results are detailed below.

## 2 Methods

### 2.1 Research object

Neonatal patients with jaundice (*n* = 78) who had received exchange transfusion therapy at the Neonatal Medical Center of our hospital between May 2017 and April 2022 were enrolled in this study as the research objects. Of these 78 patients, 45 were selected as the control group (simple exchange transfusion between 1 May 2017, and 31 December 2019), and 33 were selected as the observation group (exchange transfusion under HHFNC support between 1 January 2020, and 30 April 2022). There was no statistically significant difference between the two groups regarding general information and relevant clinical indicators (blood exchange age, weight, gender, heart rate, total bilirubin, hemoglobin, etc) (*P* > 0.05) (Tables 1, 2).

**Table 1:** Comparison of general demographic data between the two groups.

Group	Sex		Weight (kg)	Age of exchange transfusion (*d*)	Exchange transfusion time (min)	The occurrence of hemolytic diseases	
	Male	Female				Yes	No
Control	18	27	3.10 (2.98–3.70)	6.00 (4.00–7.50)	136.00 ± 8.91	13	32
Observation	17	16	3.20 (2.85–3.56)	5.00 (4.00–6.50)	133.38 ± 9.16	6	27
*χ*^2^/*t*	0.54		–	–	0.77	1.19	
*P*	0.71		0.93	0.31	0.45	0.28	

**Table 2:** Comparison of relevant clinical indicators between patients before blood exchange.

Group	Heart rate (bpm/min)	Total bilirubin (umol/L)	Hemoglobin (g/L)	Ystolic blood pressure (mmHg)	SPO_2_
Control	139.51 ± 11.97	442.68 ± 59.68	153.18 ± 22.33	442.68 ± 59.68	90.71 ± 3.45
Observation	141.18 ± 11.01	431.43 ± 61.58	140.97 ± 23.24	431.43 ± 61.58	90.58 ± 2.45
*t*	0.39	0.49	0.00	0.49	2.29
*P*	0.54	0.49	0.97	0.49	0.14

### 2.2 Inclusion criteria

The inclusion criteria were as follows: ① Full-term infant, aged <28 days; ② Infants who met the exchange transfusion criteria for neonatal jaundice, i.e., serum total bilirubin >342 pmol/L with early symptoms of bilirubin encephalopathy; ③ This study was approved by the Ethics Committee of our hospital, and the families of the patients were aware of the study aims and methods and voluntarily signed the consent form.

### 2.3 Exclusion criteria

The exclusion criteria were as follows: ① Infants with heart, liver, and kidney dysfunction; ② Infants with congenital heart disease; ③ Infants with malformations; ④ Infants with birth asphyxia and neonatal hypoxic-ischemic encephalopathy, intracranial hemorrhage, or history of neurological infection, severe cardiopulmonary disease, systemic infection, congenital malformations, and any genetic metabolic disease.

### 2.4 Methods

The neonatal hour bilirubin column chart prepared by Bhutani et al. (7) was used for determining the diagnostic and intervention criteria for neonatal hyperbilirubinemia along with the relevant standards in the “Expert Consensus on the Diagnosis and Treatment of Neonatal Hyperbilirubinemia” formulated by the Neonatology Group of the Chinese Medical Association in 2014 (8). The patients were sedated, followed by peripheral venous and arterial double-lumen synchronous exchange transfusion (selecting the radial artery and the contralateral upper or lower limb peripheral vein). Using the control group treatment, nasal intermittent humidification high-flow positive pressure ventilation, an oxygen mixer, and a suitable nasal plug were used, and the parameters were set to an inhaled oxygen concentration of 21%, a high flow rate of 4–6 L/min, and humidified inhaled gas at 36°C. The treatment was administered based on the degree of respiratory arrest. During the exchange transfusion process, simultaneous measurement of brain oxygen and intestinal oxygenation was done. After the exchange transfusion, echocardiography was done to monitor the patient's ejection fraction and pulmonary artery pressure.

#### 2.4.1 Monitoring of tissue oxygen saturation

Patients were asked to lay in the supine position, and their cerebral tissue oxygen saturation (CrSO_2_), intestinal tissue saturation (SrSO_2_), and renal tissue saturation (RrSO_2_) were determined using NIRS. The CrSO_2_ was measured by placing the B-type probe (detection depth 1 cm–1.5 cm) on the forehead (1.5 cm above the eyebrow, avoiding the midline of the brain); SrSO_2_ was measured by placing the probe 0.5 cm–1 cm below the navel; RrSO_2_ was measured by placing the probe on the side and back of the T10-L2 thoracolumbar spine (the body surface projection of the renal area); only one probe was used. The complete exchange transfusion process was done in 2 h, with 2-min interval measurements, and the average value of each index was considered.

#### 2.4.2 Salivary cortisol detection

A SARSTED saliva collection tube (Germany) was used for collecting saliva samples from patients before and 1 h after the procedure, as well as within 5 min after the end of the exchange transfusion procedure (9). The cotton swab was placed in the patient's sublingual area for 3–5 min before placing the cotton swab back into the special sleeve and tightening the cap. If the cotton swab was not placed under the tongue for the recommended 3–5 min, or if it fell out of the mouth or moved to the throat, it was quickly replaced. Saliva samples were collected without mixing with sputum or blood and stored in a freezer at −20°C. The method of collecting and preserving samples was documented a day before the procedure (10).

#### 2.4.3 MRS examination

Once the routine MRI was recorded and the images were saved, a head-specific single-channel orthogonal coil was used for the MRS examination. The ROI (region of interest) was located in the bilateral basal ganglia area on the T1WI axial image, positioned across the midline. The spectral acquisition was performed using a two-dimensional multi-voxel (MV) technique, and the pulse sequence selection was made using the point resolution spectrum (PRESS) sequence, with TR 1,500 ms and TE 144 ms. The chemical frequencies obtained from the spectral scans were processed using the Functional analysis and processing software of the GE AW 4.6 workstation. Once the voxel on one side was activated and turned green, the corresponding spectral curve was displayed. The processed data was used to calculate the peak area of metabolites, such as N-acetyl aspartate (NAA), choline (Cho), and creatine (Cr), where the peak area, ordinate, and abscissa represented the concentration, peak, chemical shift position of each metabolite. Finally, the NAA/Cr, NAA/Cho, and Cho/Cr values were calculated.1.5 Observation Indicators: The blood gas analysis (blood pH, PaCO_2_, PaO_2_), pulmonary artery pressure, ejection fraction, invasive blood pressure, heart rate, CrSO_2_, SrSO_2_, RrSO_2_, duration of exchange transfusion, concentration of salivary cortisol, NAA/Cr, NAA/Cho, and Cho/Cr were compared during the exchange transfusion process between the two groups.

### 2.5 Statistical analysis

The statistical analysis was done using the SPSS statistical software package (Version 20.0, IBM Corporation, Armonk, New York, USA). The normally distributed data was expressed as mean ± standard deviation. The group comparison was done using the paired *T*-test. The original non-normal distribution data expressed as M (Q1, Q3). Counting variables were represented as values (%). The *χ*^2^ test was used to compare between the groups. *P* < 0.05 represented statistically significant data.

## 3 Results

### 3.1 Comparison of general conditions of the two groups of patients

Table 1 shows that there was no statistical significance in gender, weight, age, the occurrence of hemolytic diseases and exchange transfusion time between the two groups of patients (*P* > 0.05).

### 3.2 Comparison of relevant clinical indicators between patients before blood exchange

Table 2 shows that there were no statistically significant differences (*P* > 0.05) in the heart rate, total bilirubin, hemoglobin, systolic blood pressure, and SPO_2_ between the two groups of patients prior to exchange transfusion.

### 3.3 Comparison of blood gas analysis, tissue oxygen saturation, hemodynamics, salivary cortisol, and brain metabolism between the two groups of patients

For the blood gas analysis, the PaCO_2_ value was significantly different between the two groups of patients (*P* < 0.05), with the observation group being lower compared with the control group. The PH and PaO_2_ values were insignificantly different (*P* > 0.05) between the two groups of patients. All relevant clinical indicators were significantly different (*P* < 0.05). For oxygenation, the CrSO_2_, SrSO_2_, and RrSO_2_ levels were significantly higher in the observation group than those in the control group (*P* < 0.05). For hemodynamic parameters, the invasive mean systolic blood pressure and ejection fraction were significantly higher in the observation group compared with those in the control group, while the pulmonary artery pressure and heart rate were significantly lower in the observation group compared with those in the control group (*P* < 0.05). The observation group had significantly lower salivary cortisol concentration compared with that in the control group (*P* < 0.05). Regarding brain metabolism, the observation group had significantly lower NAA/Cho and Cho/Cr values compared with those in the control group (*P* < 0.05) (Table 3).

**Table 3:** Comparison of blood gas analysis, tissue oxygen saturation, hemodynamics, saliva cortisol, and brain metabolism between the two groups.

Project		Control	Observation	*t*/*F*	*P*
Blood gas analysis	PH	7.41 ± 0.12	7.38 ± 0.10	0.62	0.54
	PaCO_2_	45.07 ± 5.40	40.85 ± 4.85	2.16	0.04
	PaO_2_	88.67 ± 7.09	87.92 ± 5.85	0.30	0.77
Relevant clinical indicators	Total bilirubin	225.06 ± 37.72	206.12 ± 24.67	5.30	0.02
	Hemoglobin	156.91 ± 10.37	162.52 ± 10.16	2.27	0.02
	SPO_2_	93.58 ± 2.36	95.48 ± 1.33	3.52	0.00
Tissue oxygen saturation	CrSO_2_	65.07 ± 5.78	69.92 ± 5.11	−2.34	0.027
	SrSO_2_	0.56 ± 0.08	0.62 ± 0.07	−2.11	0.045
	RrSO_2_	77.76 ± 4.23	80.13 ± 4.09	−3.02	0.016
Hemodynamics	Heart rate	153.13 ± 7.14	148.38 ± 3.64	2.16	0.04
	Mean systolic blood pressure	51.27 ± 5.08	56.54 ± 4.94	−2.77	0.01
	Pulmonary hypertension	16.8 ± 2.57	14.85 ± 1.21	2.51	0.019
	Ejection fraction	54.6 ± 4.13	59.62 ± 3.64	−2.58	0.016
Salivary cortisol	Before blood exchange	3.51 ± 0.46	3.48 ± 0.83	0.97	0.92
	Blood exchange for 1 h	3.37 ± 0.77	2.40 ± 0.67	2.99	0.008
	End of blood exchange	3.06 ± 0.55	1.71 ± 0.44	6.11	0.000
Brain metabolism	NAA/Cr	1.01 ± 0.17	0.82 ± 0.16	0.97	0.92
	NAA/Cho	0.52 ± 0.07	0.41 ± 0.05	2.99	0.008
	Cho/Cr	1.98 ± 0.23	1.71 ± 0.44	6.11	0.000

## 4 Discussion

Hyperbilirubinemia is a common disease in newborns, which can result in Bilirubin encephalopathy, which can be fatal or cause long-term neurological issues. Exchange transfusion is a treatment that can be used to reduce the risk of bilirubin encephalopathy in patients with severe hyperbilirubinemia. However, it carries significant risks and complications for the neonatal population (11, 12), such as secondary transfusion-related infections, transfusion reactions, and hemodynamic and electrolyte imbalances (13). This invasive method involves completely replacing the patient's blood with fresh blood with double circulating blood volume within a short duration, making the body's internal environment unstable, which can be high risk and stressful for patients, especially premature infants. Additional complications may include abnormal laboratory biochemical testing, low/high blood pressure, apnea, and even cardiac arrest with the probability of secondary infection (14). On the contrary, HHFNC treatment has the potential to reduce the patient's respiratory rate and improve their oxygenation (15). HHFNC has been shown to prevent airway mucosal damage and loss of moisture and heat while facilitating breathing in pediatric patients by delivering heated and heated gas (16). Physiological bilirubin possesses a strong antioxidant function; however, after a certain concentration, bilirubin gets deposited in multiple organs of the body, causing varying degrees of damage (17). Elevated bilirubin levels can cause damage to multiple organs, such as the heart, liver, and kidneys (18).

Pulse oxygen saturation (SpO_2_) can be measured through the skin to evaluate the body's oxygen supply. However, SpO_2_ only reflects the arterial oxygen saturation and does not reflect the oxygen supply to the brain. Thus, brain tissue oxygen supply abnormalities may exist even when SpO_2_ is normal (19). An abnormal increase in serum total bilirubin (TSB) levels was reported to be associated with a decrease in local cerebral oxygen saturation (CrSO_2_) (20). In newborns, the alterations in the TSB levels before and after blood transfusion (BET) can affect various factors, including blood flow metabolism, brain metabolism, cerebral blood flow, and systemic oxygenation. All of these can affect CrSO_2_ (21, 22). A decrease in brain metabolism decreases the oxygen uptake, increasing CrSO_2_ (21, 22). The changes in CrSO_2_ indicate a balance between new perfusion and neuronal damage (23). Children with severe hyperbilirubinemia are at risk of suffering from neuronal damage due to the sustained central toxicity to the brain caused by the rapid increase in TSB. Thus, it is essential to consider the impact of TSB on CrSO_2_ (24). In this study, the oxygen uptake of the patients was improved by providing HHFNC, avoiding complications caused by cerebral tissue hypoxia during blood exchange. An important indicator of local tissue blood perfusion and oxygen metabolism is intestinal tissue oxygen saturation. Due to insufficient blood flow, the intestinal blood flow is poorly regulated, weakening the oxygen-carrying capacity of intestinal tissue and causing potential intestinal ischemia. This considerably increases the risk of transfusion-related necrotizing enterocolitis. The blood gas analysis results indicated that the acid-base balance of the observation group was not disturbed.

Due to inadequate ventilation, the control group's carbon dioxide partial pressure of the control group was slightly higher than the average value. HHFNC improved the internal environment disorder caused by inadequate ventilation. The results of tissue oxygen saturation results suggested that the observation group exhibited better oxygenation in the brain, intestines, and kidneys. This helped prevent any complications arising from cerebral tissue hypoxia during blood exchange. Early correction of hypoxia in brain, intestinal, and renal tissues considerably reduced the damage caused by hypoxia. Near-infrared spectroscopy (NIRS) can be used to directly measure the oxygen metabolism levels in the brain, intestines, and kidneys oxygen metabolism at a given moment. This continuous real-time monitoring based on the fluctuation of the measured data facilitates the evaluation of the patient's oxygen supply and demand levels. NIRS has been reported to show real-time changes in CrSO_2_ (24). The results of this study showed that using HHFNC during blood exchange can reduce bilirubin levels, increase hemoglobin levels, and improve heart rate, SPO_2_, and blood pressure in children. During the blood exchange process, it was found that there were fluctuations in the level of CrSO_2_ before and after BET. CrSO_2_ showed a slow upward trend during the BET process, which may increase the protein binding ability of blood to free bilirubin with fresh plasma, reduce the level of bilirubin in the body, and improve the function of hemoglobin to transport oxygen; After lowering the bilirubin levels, there is a reduction in bilirubin neurotoxicity, leading to an improvement in cerebral perfusion, consistent with the results reported by Lu Chunmei et al. (24). HHFNC produces a high-speed humidified airflow through the nasal cavity, which generates a pressure of about 2.7 cm H_2_O in the respiratory tract. This is similar to the effect of positive end-expiratory pressure ventilation. This positive pressure reduces anatomical dead space, improves patient oxygenation, reduces respiratory effort, and improves lung function. The normal functions of the brain rely heavily on aerobic metabolism (19), implying that brain injury is closely related to the oxygen supply of brain tissue. The hemodynamic results indicated that the observation group exhibited higher invasive blood pressure, ejection fraction, and pulmonary artery pressure than the control group. HHFNC affects the heart and circulatory system by reducing thoracic negative pressure, venous return, cardiac output, blood pressure, and circulating blood volume (25). It also reduces adverse reactions that may occur during blood exchange treatment. Effective treatment was given promptly to avoid irreversible damage to the nervous system of the patients (26, 27) and had a particular significance in improving the cardiac hemodynamics of the patients. The hypothalamic pituitary adrenal axis is an important endocrine axis in the human body, with cortisol as its main product. The functional status of the HPA axis and cortisol levels are indicators of the human body's ability to adapt to the environment and respond to stimuli (9). In this study, the salivary cortisol concentration index indicates the adverse stimulation response of pediatric patients. During the blood exchange process, high-flow ventilation was employed to increase the comfort of pediatric patients and reduce their stress response.

Magnetic resonance spectroscopy (MRS) can effectively detect small changes in the peak values of nuclear protons of the above metabolites in brain tissue. This method can be used to determine the metabolic status of brain tissue cells and evaluate the patient's condition more accurately and comprehensively. The results of brain metabolism analysis reveal that a decrease in the NAA/Cr and Cho/Cr ratios is associated with a lower chance of brain injury. Improving the oxygenation of the patients can reduce the impact on the brain cells and reduce the risk of brain tissue damage. The increase in NAA/Cr and Cho/Cr ratios can result in metabolic abnormalities after brain tissue damage. Conversely, a reduction in the NAA/Cr ratio indicates no brain damage. Persistent bilirubin neurotoxicity can cause abnormal development of the cerebellum, neuronal cell death, and circulatory metabolic disorders in the brain. These conditions can lead to significant fluctuations in brain oxygen levels. The findings of this study reveal a statistically significant difference in CrSO_2_ levels before and after BET. Real-time monitoring of CrSO_2_ using NIRS can effectively detect changes in brain perfusion during BET, reducing the occurrence of hypoxic brain injury, consistent with the results reported by Lu Chunmei et al. (24).

## 5 Conclusions

The findings of this study revealed that HHFNC provided tolerable ventilation pressure support for pediatric patients and the tissue oxygen saturation increased during the blood exchange process, leading to fluctuations in CrSO_2_, SrSO_2_, and RrSO_2_. This increase in saturation helps maintain blood flow stability and organ oxygenation, reducing adverse reactions caused by blood exchange, minimizing stress in children, and reducing the impact on brain metabolism.

## Data Availability

The original contributions presented in the study are included in the article/Supplementary Material, further inquiries can be directed to the corresponding author.
